# Supplementary material for: Regulation of Glutathione S-Transferase Omega 1 Mediated by Cysteine Residues Sensing the Redox Environment
Source: Int J Mol Sci. 2024 May 12;25(10):5279. doi: 10.3390/ijms25105279 (PMC11121155; doi:10.3390/ijms25105279)
Supplement: Supplementary file 1 [file ijms-25-05279-s001.zip › ijms-2985222-supplementary.pdf]

Supplementary Table S1. Primers for site-directed mutagenesis.

GstO1 mutants		Sequence (5'-3')
C32A	Forward	GTC TAC AGC ATG AGG TTC GCG CCC TTC GCT CAG AG
	Reverse	CTC TGA GCG AAG GGC GCG AAC CTC ATG CTG TAG AC
C90A	Forward	CGA ATC TGT CAT CAC TGC GGA GTA CCT GGA TGA GG
	Reverse	CCT CAT CCA GGT ACT CCG CAG TGA TGA CAG ATT CG
C191A	Forward	GAA GCA TTG GAG CTC AAG GAG GCG CTA GCC CAC AC
	Reverse	GTG TGG GCT AGC GCC TCC TTG AGC TCC AAT GCT TC
C236A	Forward	GCC CCG AGG CCG CAG ATT ATG GGC TC
	Reverse	GAG CCC ATA ATC TGC GGC CTC GGG GC
